# Species-Specific Detection and Identification of *Fusarium* Species Complex, the Causal Agent of Sugarcane Pokkah Boeng in China

**DOI:** 10.1371/journal.pone.0104195

**Published:** 2014-08-20

**Authors:** Zhenyue Lin, Shiqiang Xu, Youxiong Que, Jihua Wang, Jack C. Comstock, Jinjin Wei, Per H. McCord, Baoshan Chen, Rukai Chen, Muqing Zhang

**Affiliations:** 1 Guangxi University, Nanning, Guangxi, China; 2 Fujian Agriculture and Forestry University, Fuzhou, China; 3 USDA-ARS-Sugarcane Field Station, Canal Point, Florida, United States of America; St. Petersburg Pasteur Institute, Russian Federation

## Abstract

**Background:**

Pokkah boeng disease caused by the *Fusarium* species complex results in significant yield losses in sugarcane. Thus, the rapid and accurate detection and identification of the pathogen is urgently required to manage and prevent the spreading of sugarcane pokkah boeng.

**Methods:**

A total of 101 isolates were recovered from the pokkah boeng samples collected from five major sugarcane production areas in China throughout 2012 and 2013. The causal pathogen was identified by morphological observation, pathogenicity test, and phylogenetic analysis based on the fungus-conserved rDNA-ITS. Species-specific TaqMan real-time PCR and conventional PCR methods were developed for rapid and accurate detection of the causal agent of sugarcane pokkah boeng. The specificity and sensitivity of PCR assay were also evaluated on a total of 84 isolates of *Fusarium* from China and several isolates from other fungal pathogens of *Sporisorium scitamineum* and *Phoma* sp. and sugarcane endophyte of *Acremonium* sp.

**Result:**

Two *Fusarium* species (*F. verticillioides* and *F. proliferatum*) that caused sugarcane pokahh boeng were identified by morphological observation, pathogenicity test, and phylogenetic analysis. Species-specific TaqMan PCR and conventional PCR were designed and optimized to target their rDNA-ITS regions. The sensitivity of the TaqMan PCR was approximately 10 pg of fungal DNA input, which was 1,000-fold over conventional PCR, and successfully detected pokkah boeng in the field-grown sugarcane.

**Conclusions/Significance:**

This study was the first to identify two species, *F. verticillioides* and *F. proliferatum*, that were causal pathogens of sugarcane pokkah boeng in China. It also described the development of a species-specific PCR assay to detect and confirm these pathogens in sugarcane plants from mainland China. This method will be very useful for a broad range of research endeavors as well as the regulatory response and management of sugarcane pokkah boeng.

## Introduction

Sugarcane pokkah boeng is an economically important fungal disease worldwide [1], which was first described in Java by Walker and Went in 1896, and the name was a Javanese term denoting a malformed or distorted top. Since then, pokkah boeng has been recorded in almost all cane growing countries, but it only causes severe damage in areas where susceptible varieties are widely planted during a hot and dry season followed by a wet season [2]. A survey of different sugarcane areas in India found that the incidence of pokkah boeng increased from 2007 to 2013 and affected almost all of the sugarcane cultivars, which was recommended for general cultivation for different agricultural climatic regions. From 2012 to 2013, a 90% infection rate was observed in S224/20 from Shahjahanpur, India, while CoSe01434 had a 5%–30% infection rate [3].

Three to seven month-old sugarcane is more susceptible to infection than plants in later stages of growth [1]. After infection, the leaves become crumpled, twisted, and shortened. Irregular reddish stripes and specks then develop within the chlorotic tissue and form lens or rhomboid-shaped holes. Leaf sheaths may also become chlorotic and develop irregular necrotic areas of reddish color [1]. The most serious injury is infection of the growing tip of the plant, which results in the loss of the entire top of plant and is referred to as top rot [1], [4]. Thus, sugarcane pokkah boeng has become a serious threat to sugarcane production in China. In addition, the incidence and severity of pokahh boeng has been reported from major sugarcane growing areas during all seasons, rather than only during the wet and hot summer seasons.

Pokkah boeng is caused by the *Fusarium* species complex, but it is not known which of the large number of species contribute to disease outbreaks. One pathogen responsible for pokkah boeng was first described as *Gibberella fujikuroi* (Sawada) in 1904 [5]. In South Africa, *F. sacchari, F. proliferatum,* and *F. andiyazi* were identified as causal agents as a result of inoculation experiments in potted plants [6]. Forty-one isolates of *F. verticillioides* or *F. subglutinans* produced symptoms of pokkah boeng [7]. In addition, sugarcane stalk wilt and rot caused by *F. sacchari*, which was closely related to pokkah boeng caused by *F. moniliforme* (*F. verticillioides*), occasionally occurred on stems close to the top of plants [5]. However, to date, the species of *Fusarium* species complex that cause sugarcane pokahh boeng in China has not been identified.

Disease management strategies require not only the detection and identification of destructive pathogens, but also an understanding of the threshold pathogen density, changes in the distribution of the pathogen, and the interaction between the pathogen and its biotic and abiotic environments [8]. Conventional PCR is widely used in pathogen identification, mostly due to the low cost of thermal cyclers and reagents, while TaqMan real-time PCR (TaqMan PCR) has the advantages of speed, sensitivity, and the ability to quantify pathogens [9]. The rDNA internal transcribed spacer (ITS) sequences have been successfully used in resolving species-level and phylogenetic relationships in *Fusarium* [10]–[12]. In this study, we developed a species-specific method to detect and identify the species of *Fusarium* species complex isolated from all of the major sugarcane production areas throughout the year that caused sugarcane pokahh boeng in China.

## Materials and Methods

### 2.1 Fungal isolates

Samples of sugarcane plants with pokkah boeng symptoms were observed and collected from five major sugarcane production areas in China (Guangxi, Yunnan, Guangdong, Fujian and Hainan, location of sample areas see Fig. S1) throughout 2012 and 2013. No any specific permission was required for these locations/activities. The field studies did not involve endangered or protected species. A total of 101 isolates were recovered from the collected samples (see Table S1). Leaf tissue (5×5 mm) cut from the margins of diseased sections was surface-sterilized by dipping in 70% ethanol for 10 s, followed by 30 s in 0.1% HgCl_2_ solution. They were then rinsed three times in sterile water, placed on potato dextrose agar (PDA) medium, and incubated in darkness at 28°C. Single spore cultures were derived and maintained on PDA and their identity was confirmed by morphological characterization using standard protocols [13]. Two related isolates of *F. fujikuroi* (EM-1-10 and EM-1-17, gifts from Zhejiang Univ.) from bakanae disease of rice, and two sugarcane isolates each from *Phoma* sp. (FJ1 and FJ2, pathogen causing twisting and curling of crown sugarcane leaves collected in Guangxi, China), *Sporisorium scitamineum* (S201301 and S201302, pathogen of sugarcane smut disease collected in Fujian, China) and *Acremonium* sp. (A1 and A2, sugarcane endophyte collected in Guangxi, China), were used for comparisons in this study.

### 2.2 Pathogenicity test

Conidial suspensions (10^7^ CFU/ml) of the isolates from CNO-1 and YN-41 were micro-injected into 20 sugarcane seedlings of cultivar ROC22. An additional 20 seedlings were injected with water without conidia as a control. The inoculated plants were grown in a growth-chamber at 28°C with a 16 h photoperiod. Symptoms were observed on the inoculated leaves at 8 d post-inoculation. The fungus was re-isolated and identified as described in section 2.1.

### 2.3 DNA extraction, ITS-PCR and sequencing

The isolates were transferred to potato dextrose water (PDW) and grown in shaken cultures for 4 days at 28°C. Total DNA was extracted from fungal mycelia using the SDS method with minor modifications [14]–[15]. Briefly, mycelia were suspended in 300 µl of lysis buffer (100 mM Tris-HCl, 10 mM EDTA pH 8, 2% Triton X-100, 1% SDS and 100 mM NaCl) and 300 µl of phenol:chloroform:isoamyl alcohol (25∶24∶1) and then vortexed to release DNA. After centrifugation at 3,000×g for 5 min, the supernatant was mixed with 300 µl of chloroform:isoamyl alcohol (24∶1) and then centrifuged again. The pellet was washed with 70% ethanol, dried, re-suspended in 100 µl ddH_2_O, and stored at −20°C for further use. The yield and purity of DNA were determined by spectrophotometry (Thermo Scientific Multiskan GO, USA). The internal transcribed spacer (ITS) of rDNA was amplified using fungus-conserved primer sequences ITS1 (TCC,GTA,GGT,GAA,CCT,GCG,G) and ITS4 (TCC,TCC,GCT,TAT,TGA,TAT,GC) [16]. Each amplification reaction included 25 µl of Dream Taq Green PCR Master MIX (Thermo Fisher Science Inc. California), 5 µl (10 µM) of each primer, and 14 µl of ddH_2_O in a final volume of 50 µl. The amplification program was as follows: an initial denaturation step of 5 min at 94°C, followed by 35 cycles consisting of 1 min at 94°C, 1 min at 56°C, and 3 min at 72°C, and a final elongation step of 10 min at 72°C. PCR reactions were performed on a GeneAmp PCR System 9700 (Applied Biosystems, Foster City, CA). The amplified products were sequenced by Sangon, Shanghai (Sangon Biotech Co., Ltd. Shanghai, China).

### 2.4 Sequence assembly, alignment, and phylogenetic trees

The obtained sequences were first analyzed with the alignment program provided in the DNAMAN version 4.0 (DNAMAN, Lynnon Biosoft, USA). After sequence assembly, the complete sequences of each ITS fragment (partial sequence of 18S ribosomal RNA gene; complete sequence of internal transcribed spacer 1; 5.8S ribosomal RNA gene; internal transcribed spacer 2; and partial sequence of 28S ribosomal RNA gene) were deposited in GenBank with accession numbers from KJ629468 to KJ629570 (see Table S1). For phylogenetic analysis of the *Fusarium* species complex, reference DNA-ITS sequences of known *Fusarium* isolates were retrieved from the fungal biodiversity center (CBS) and the GenBank in National Center for Biotechnology Information (NCBI) (see Table S1). Nucleotide sequences were aligned using CLUSTALW (v 1.6) present in MEGA 6.0, followed by manual modification [17]. Aligned nucleotide sequences were used to construct phylogenetic trees using the MEGA (v 6.0) software package [18]. Phylogenetic trees were constructed based on the neighbor-joining (NJ) and Kimura 2-parameter method. Bootstrap resampling (1,000 replications) was used to measure the reliability of individual nodes in the phylogenetic tree [17], [19].

### 2.5 Species-specific primer and probe design, and PCR program

Two species-specific primers and probes for TaqMan PCR were designed based on the ITS region of rDNA gene sequence from the above identified *Fusarium* species based on morphological and molecular characterization. The primer-probe combinations denoted FAM-gx1-F/FAM-gx1-R/FAM-gx1-P (FAM-gx1) and FAM-gx2-F/FAM-gx2-R/FAM-gx2-P (FAM-gx2) were specific to the identified species, respectively. The TaqMan probes were labeled at the 5′-terminal nucleotide with 6-carboxy-fluorescein (FAM) reporter dye and at the 3′-terminal nucleotide with Black Hole Quencher (BHQ)-1. In the conventional PCR, two pairs of primers, denoted Pgx1-F/Pgx1-R (Pgx1) and Pgx2-F/Pgx2-R (Pgx2), were also designed based on the same ITS region of rDNA gene sequence for species-specific amplification of the identified species, respectively. A BLASTn query against the NCBI GenBank database was used to ensure the specificity of the primers and probes prior to synthesis by Integrated DNA Technologies, Inc. (Coralville, Iowa USA).

TaqMan PCR amplifications were performed using a Roche Light Cycler 480 (Indianapolis, IN, USA) in a 20 µl reaction volume consisting of the following reagents at the optimized concentrations: 500 nM each primer (FAM-gx1-F/FAM-gx1-R or FAM-gx2-F/FAM-gx2-R), 250 nM probe (FAM-gx1-P or FAM-gx2-P), 10 µl Premix LA Taq (TaKaRa Biotechnology, Dalian, China), and 9 µl of ddH_2_O. The amplification protocol was 95°C for 30 s followed by 40 cycles at 95°C for 5 s and 60°C for 30 s. Each run included one negative (healthy plant) and one positive control (diseased plant). The data were analyzed using the Light Cycler ®480 software.

Conventional PCR, including one of each primer set Pgx1-F/Pgx1-R or Pgx2-F/Pgx2-R, was performed in a 25 µl PCR reaction mixture. Each amplification reaction included 0.1 µl of Taq DNA Polymerase (5 U/µl) (Thermo Fisher Science Inc. GO, USA), 2.5 µl of Taq buffer (10×), 1 µl of MgCl_2_ (25 mM), 1 µl of each primer set (10 µM), 0.4 µl of dNTPs (10 mM/each), 1 µl of sample DNA template, and 18 µl of ddH_2_O. Amplification conditions for both regions consisted of an initial denaturation step of 5 min at 94°C, followed by 30 cycles of 94°C for 30 s, 63°C for 15 s, and 72°C for 30 s, and a final extension step of 10 min at 72°C. All PCR reactions were performed on a Mastercycler® Pro (Hauppauge, NY, USA), followed by electrophoresis on 1.0% agarose gels. PCR products were visualized by staining with GelRed reagent (Biotium, Hayward, CA).

### 2.6 Evaluation of the PCR assays

The specificity of TaqMan PCR and conventional PCR was evaluated using DNA extracted from 84 isolates from China as well as DNA extracted from *Sporisorium scitamineum*, *Phoma* sp. and *Acremonium* sp. A 10-fold serial dilution of extracted DNA from the isolates of CNO-1 and YN-41 was quantified for evaluation of the sensitivity of TaqMan PCR and conventional PCR.

### 2.7 Pathogen detection of pokahh boeng from field-grown sugarcane

The multiplex TaqMan PCR was used to detect the pathogen of pokahh boeng in the field-grown sugarcane plants. Leaves were sampled from 28 field-grown sugarcane plants in Nanning, Chongzuo, and Hechi, Guangxi Zhuang autonomous regions during October 2013, among which 5 were asymptomatic (As) and 23 were symptomatic (S) for pokkah boeng (Table 1). These samples were tested with TaqMan PCR and conventional PCR, respectively. A positive internal control primer–probe set was designed to assess the quality of sugarcane plant DNA based on the sequence of the conserved 25S *rDNA* (Genbank accession No. CO373883) [20], [21].

**Table 1:** TaqMan PCR and conventional PCR for detection of asymptomatic (As) and symptomatic (S) pokkah boeng leaves from the field-grown sugarcane.

Samples	TaqMan real-time PCR w		Conventional PCR y		Internal control 25S *rDNA*
	FAM-gx1	FAM-gx2	Pgx1	Pgx2	
As1	0	0	−	−	**+**
As2	0	0	−	−	**+**
As3	0	0	−	−	**+**
As4	0	0	−	−	**+**
As5	0	0	−	−	**+**
S1	24.88	0	−	−	**+**
S2	28.64	27.80	−	−	**+**
S3	26.32	0	−	−	**+**
S4	19.86	0	**+**	−	**+**
S5	27.73	25.93	−	−	**+**
S6	29.19	29.90	−	−	**+**
S7	22.43	0	−	−	**+**
S8	27.07	0	−	−	**+**
S9	28.22	29.57	−	−	**+**
S10	28.52	0	−	−	**+**
S11	28.30	0	−	−	**+**
S12	26.53	0	−	−	**+**
S13	27.63	29.75	−	−	**+**
S14	30.82	29.73	−	−	**+**
S16	26.20	0	−	−	**+**
S17	28.35	0	−	−	**+**
S21	0	0	−	−	**+**
S22	29.81	0	−	−	**+**
S23	22.71	0	−	−	**+**
S25	28.49	0	−	−	**+**
S26	27.83	0	−	−	**+**
S27	28.36	0	−	−	**+**
S28	21.58	26.99	−	−	**+**
NTC^z^	0	0	−	−	−
NTC	0	0	−	−	−

w For quantitative TaqMan PCR reactions, two primer-probe combinations were used; FAM-gx1 is specific to *F. verticillioides*; FAM-gx2 specific to *F. proliferatum*.

y For conventional PCR, two primer sets were used; Pgx1 is specific to *F. verticillioides*; Pgx2 specific to *F. proliferatum*. ‘+’ is positive; ‘−’ is negative.

z NTC: Negative test control (water).

## Results

### 3.1. Species identification of the *Fusarium* isolates using phylogenetic analysis and morphological observation

**Figure 1:**
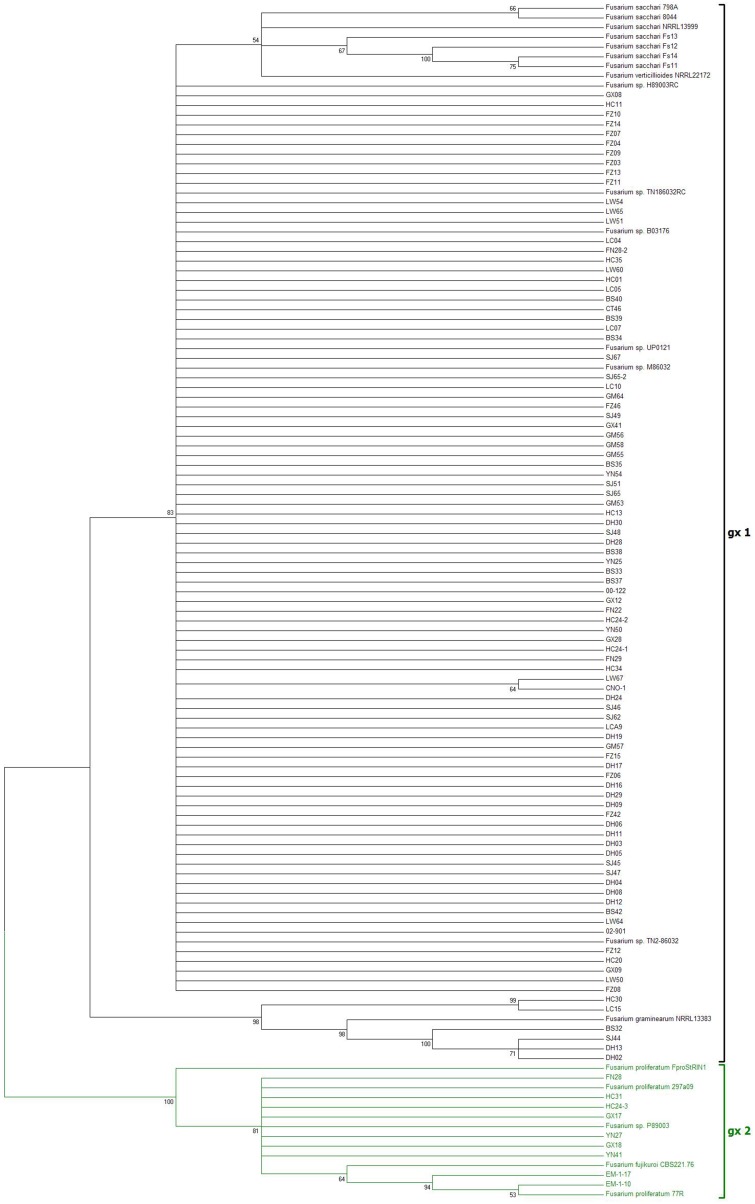
Phylogenetic trees of 103 isolates based on ITS-rDNA sequence amplified by fungus-conserved ITS1 and ITS4. A phylogenetic tree was constructed on the 101 sugarcane isolates of *Fusarium* species complex from China, and 2 isolates from rice bakanae disease. The reference sequences were retrieved from the fungal Biodiversity center (CBS) and the GenBank in National Center for Biotechnology Information (NCBI). The phylogenetic tree was constructed by the neighbor-joining (NJ) and Kimura 2-parameter method with bootstrap resampling (1,000 replications) using the aligned nucleotide sequences of ITS-rDNA. Numbers above the nodes are bootstrap values (>50%) from 1,000 replicates.

The sequences of the internal transcribed spacer from 103 isolates were amplified by PCR using fungus-conserved ITS1 and ITS4 primers, which resulted in a fragment in the size range of 485 bp to 603 bp. The PCR products were used for sequencing directly. The sequence alignment indicated that there were diversity and differences among the isolates of *Fusarium* species complex studied. The phylogenetic tree was constructed using neighbor-joining (NJ) method whereas some known ITS sequences from *Fusarium* species complex (including *F. verticillioides, F. sacchari, F. proliferatum*, *F. graminearum* and *F. fujikuroi*) were used as references for the out-group (Fig. 1). The phylogenetic analysis indicated that all 103 isolates were clustered into two groups with 100% of bootstraps, the first phylogenetic group (gx1) included 94 sugarcane isolates (93%) and some reference sequences from *F. verticillioides* NRRL22172 and *F. sacchari* NRRL13999; the second phylogenetic group (gx2) included only 7 sugarcane isolates (7%) and isolates of EM-1-10 and EM-1-17 as well as some reference sequences from *F. proliferatum* and *F. fujikuroi*.

**Figure 2:**
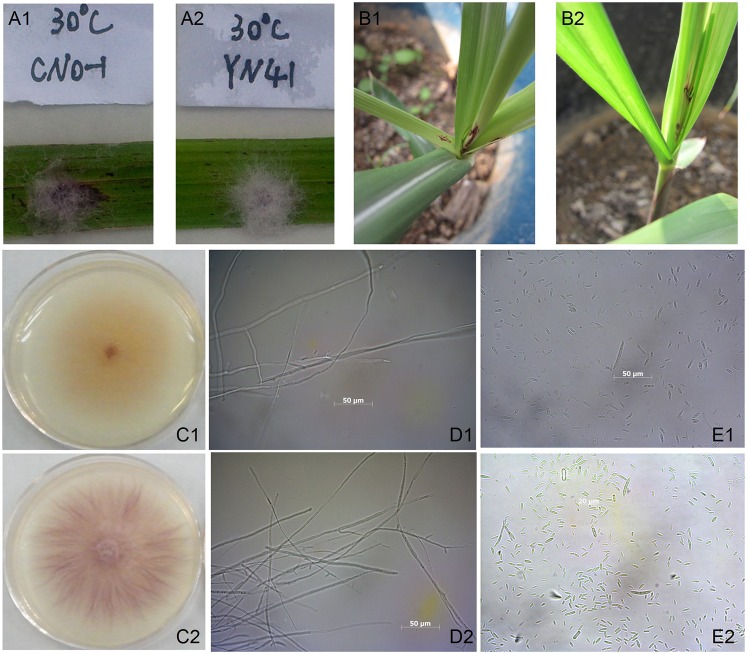
Morphological observation and pathogenicity test of the isolates of *F. verticillioides* and *F. proliferatum* (1: CNO-1 and 2: YN-41). (A) Fungal growth on the leaf; (B) Inoculated plants at 8 d after micro-injection; (C) Colony on potato dextrose agar; (D) Hyphae at 400×; (E) Conidia at 400×.

The fungal colony of the CNO-1 isolate (gx1) appeared to be pale in color but became orange at the top as it aged, while it was initially white at the bottom which later changed into a yellow color. Macroconidia with two to four-septate were observed in size ranged from 10–45×1.5–4.1 µm and were falcate and curved, slender and dorsiventral. Abundant microconidia were also observed with sizes ranging from 7.1–13.4×0.5–2.1 µm and were clavateor ellipsoidal as well as septate or mostly aseptate. Conidia adhered only in the false heads, but chains and sporodochial conidia were absent. Conidiophores were prostrate and mostly branched. However, the colony morphology of YN-41 (gx2) was greatly different from that of CNO-1 (gx1). From the top view, the color of the fungal colony was violet or brownish-red while the color was white and cream at the bottom. Macroconidia were slender, slightly falcate, two to six-septate, 18.5–42.5×1.1–2.3 µm with slightly curved apical cell produced on cream to orange sporodochia. Microconidia were single-celled, oval, 8.1–12.6×1.15–4 µm and produced on mono- and polyphialides in false heads and in short chains. Pyriform and clavate conidia were produced in chains and in false heads. Chlamydospores were absent, but sporodochia were present (Fig. 2).

The typical symptoms of pokahh boeng seen in the field, such as chlorotic tissue and lens or rhomboid-shaped holes, appeared on the inoculated leaves at 8 d after inoculation, while the control leaves remained asymptomatic (Fig. 2). Based on the morphological criteria presented in the *Fusarium* Laboratory Manual [13] and molecular phylogenetic analysis, the pathogens of sugarcane pokahh boeng had two species. The first phylogenetic group (gx1) was the dominant species (accounting for over 90%), which belonged to *F. verticillioides* and was closely related to *F. sacchari*, while the second phylogenetic group (gx2) was the minor species (less than 10%), which belonged to *F. proliferatum*, and was closely related to *F. fujikuroi*.

### 3.2 Specificity and sensitivity of TaqMan PCR and conventional PCR

**Figure 3:**
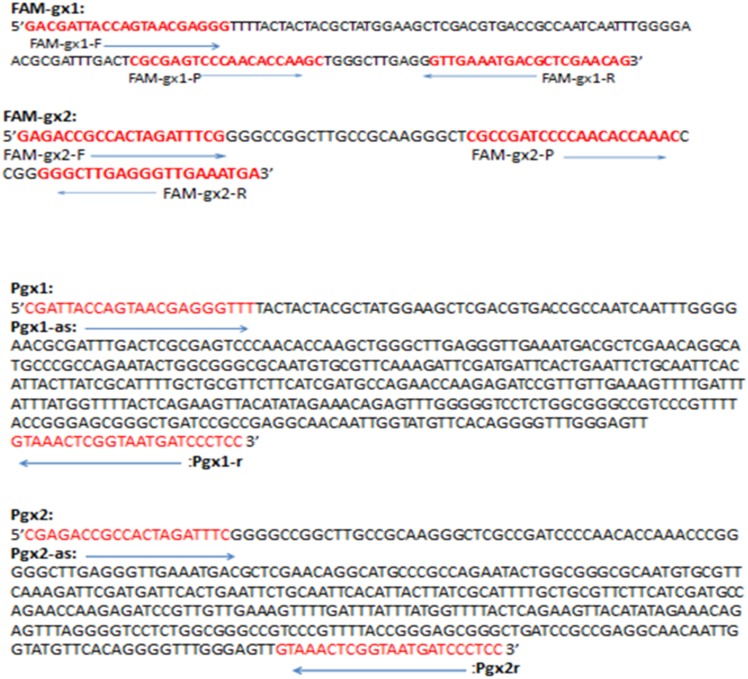
Species-specific primer and probe sequences for amplification of *F. verticillioides* and *F. proliferatum* based on the aligned rDNA-ITS sequence. (A) primer–probe combinations for TaqMan real-time PCR assay: FAM-gx1 is specific to *F. verticillioides*; FAM-gx2 is specific to *F. proliferatum*; (B) Primer sets for conventional PCR assay: Pgx1 is specific to *F. verticillioides*; Pgx2 is specific to *F. proliferatum*.

A nucleotide sequence alignment of the ITS1-rDNA region revealed significant differences between gx1 (*F. verticillioides*) and gx2 (*F. proliferatum*). Based on these nucleotide differences in sequence, primer-probe combinations for TaqMan PCR assay, FAM-gx1-F/FAM-gx1-P/FAM-gx1-R (FAM-gx1) and FAM-gx2-F/FAM-gx2-P/FAM-gx2-R (FAM-gx2), as well as the primer pair sets for conventional PCR, Pgx1-F/Pgx1-R (Pgx1) and Pgx2-F/Pgx2-R (Pgx2), were designed to be specific to the species of *F. verticillioides* (gx1) and *F. proliferatum* (gx2), respectively (Fig. 3). Specificity of the above PCR was evaluated from seventy-eight isolates from gx1 (*F. verticillioides*) and six isolates from gx2 (*F. proliferatum*) while two isolates each of *Phoma* sp. (FJ1 and FJ2), *Sporisorium scitamineum* (S201301 and S201302), and *Acremonium* sp. (A1 and A2) from sugarcane were used as controls. The TaqMan PCR using FAM-gx1 yielded positive results (Ct value ranged from 14.8 to 30.3) from 78 isolates of *F. verticillioides* (gx1), while negative results (Ct values of zero or undetectable) were obtained from 6 isolates of *F. proliferatum* (gx2) and the other fungal controls. The assays using FAM-gx2 produced positive results (Ct value ranged from 17.2 to 24.3) only from 6 isolates of *F. Proliferatum* (gx2), while all the others tested negative (Ct values of zero or undetectable). Results from the conventional PCR indicated that the species-specific PCR product at the predicted size of 439 bp was amplified using Pgx1 in the same 78 isolates of *F. verticillioides* and a 400 bp fragment was amplified using Pgx2 in the other 6 isolates of *F. proliferatum.* The other fungal controls (isolates from *Sporisorium scitamineum, Phoma* sp., and *Acremonium* sp.) tested negative using both primer sets of Pgx1 or Pgx2 (Table 2).

**Table 2:** Specificity of TaqMan PCR and conventional PCR for *F. verticillioides, F. proliferatum* and other sugarcane fungal pathogens (*Sporisorium scitamineum* and *Phoma* sp.*)* and endophyte (*Acremonium* sp.).

Isolates	Species	TaqMan PCR (FAM Ct values)		Conventional PCR	
		FAM-gx1	FAM-gx2	Pgx1	Pgx2
DH02	*F. verticillioides*	28.5	0	+	−
DH03	*F. verticillioides*	20.78	0	+	−
DH04	*F. verticillioides*	18.88	0	+	−
DH06	*F. verticillioides*	20.42	0	+	−
DH09	*F. verticillioides*	27.3	0	+	−
DH11	*F. verticillioides*	22.05	0	+	−
DH12	*F. verticillioides*	21.88	0	+	−
DH13	*F. verticillioides*	29.27	0	+	−
DH16	*F. verticillioides*	14.77	0	+	−
DH17	*F. verticillioides*	19.76	0	+	−
DH19	*F. verticillioides*	18.27	0	+	−
DH24	*F. verticillioides*	16.52	0	+	−
YN25	*F. verticillioides*	18.49	0	+	−
DH28	*F. verticillioides*	19.41	0	+	−
DH29	*F. verticillioides*	17.21	0	+	−
DH30	*F. verticillioides*	16.86	0	+	−
BS32	*F. verticillioides*	29.41	0	+	−
BS33	*F. verticillioides*	19.33	0	+	−
BS34	*F. verticillioides*	16.81	0	+	−
BS35	*F. verticillioides*	18.72	0	+	−
BS37	*F. verticillioides*	17.17	0	+	−
BS38	*F. verticillioides*	16.21	0	+	−
BS39	*F. verticillioides*	18.17	0	+	−
BS42	*F. verticillioides*	16.21	0	+	−
SJ44	*F. verticillioides*	30.29	0	+	−
SJ45	*F. verticillioides*	19.12	0	+	−
SJ46	*F. verticillioides*	20.72	0	+	−
SJ47	*F. verticillioides*	17.17	0	+	−
SJ48	*F. verticillioides*	20.44	0	+	−
SJ49	*F. verticillioides*	16.95	0	+	−
YN50	*F. verticillioides*	18.64	0	+	−
SJ51	*F. verticillioides*	16.99	0	+	−
GM53	*F. verticillioides*	22.53	0	+	−
YN54	*F. verticillioides*	18.67	0	+	−
GM55	*F. verticillioides*	20.69	0	+	−
GM56	*F. verticillioides*	15.87	0	+	−
GM57	*F. verticillioides*	24.25	0	+	−
GM58	*F. verticillioides*	19.33	0	+	−
SJ62	*F. verticillioides*	15.94	0	+	−
GM64	*F. verticillioides*	21.15	0	+	−
SJ65	*F. verticillioides*	17.82	0	+	−
SJ67	*F. verticillioides*	16.94	0	+	−
CT46	*F. verticillioides*	23.48	0	+	−
HC01	*F. verticillioides*	17.56	0	+	−
GX08	*F. verticillioides*	16.64	0	+	−
HC11	*F. verticillioides*	19.15	0	+	−
GX12	*F. verticillioides*	22.22	0	+	−
HC13	*F. verticillioides*	21.05	0	+	−
HC20	*F. verticillioides*	22.12	0	+	−
HC24-1	*F. verticillioides*	17.72	0	+	−
HC24-2	*F. verticillioides*	17.88	0	+	−
HC30	*F. verticillioides*	20.98	0	+	−
HC34	*F. verticillioides*	16.17	0	+	−
HC35	*F. verticillioides*	23.23	0	+	−
GX28	*F. verticillioides*	24.5	0	+	−
LC04	*F. verticillioides*	19.43	0	+	−
LC07	*F. verticillioides*	20.72	0	+	−
GX09	*F. verticillioides*	22.67	0	+	−
LC10	*F. verticillioides*	19.73	0	+	−
LC15	*F. verticillioides*	20.08	0	+	−
LW50	*F. verticillioides*	22.95	0	+	−
LW51	*F. verticillioides*	21.27	0	+	−
LW64	*F. verticillioides*	20.01	0	+	−
LW65	*F. verticillioides*	24.5	0	+	−
LW67	*F. verticillioides*	20.79	0	+	−
GX41	*F. verticillioides*	21.68	0	+	−
FZ03	*F. verticillioides*	19.79	0	+	−
FZ04	*F. verticillioides*	21.84	0	+	−
FZ06	*F. verticillioides*	20.64	0	+	−
FZ07	*F. verticillioides*	21.22	0	+	−
FZ08	*F. verticillioides*	21.84	0	+	−
FZ09	*F. verticillioides*	17.23	0	+	−
FZ10	*F. verticillioides*	19.23	0	+	−
FZ11	*F. verticillioides*	19.46	0	+	−
FZ12	*F. verticillioides*	17.86	0	+	−
FZ13	*F. verticillioides*	21.15	0	+	−
FZ15	*F. verticillioides*	18.58	0	+	−
CNO-1	*F. verticillioides*	18.07	0	+	−
YN41	*F. proliferatum*	0	17.16	−	+
HC31	*F. proliferatum*	0	19.88	−	+
HC24–3	*F. proliferatum*	0	20.89	−	+
YN27	*F. proliferatum*	0	20.9	−	+
GX18	*F. proliferatum*	0	23.66	−	+
GX17	*F. proliferatum*	0	24.25	−	+
FJ-1	*Phoma* sp.	0	0	−	−
FJ-2	*Phoma* sp.	0	0	−	−
S201301	*S. scitamineum*	0	0	−	−
S201302	*S. scitamineum*	0	0	−	−
A-1	*Acremonium* sp.	0	0	−	−
A-2	*Acremonium* sp.	0	0	−	−

For quantitative TaqMan PCR reactions, two primer-probe combinations were used; FAM-gx1 is specific to *F. verticillioides*; FAM-gx2 specific to *F. proliferatum*. For conventional PCR, two primer sets were used; Pgx1 is specific to *F. verticillioides*; Pgx2 specific to *F. proliferatum*. ‘+’ is positive; ‘−’ is negative.

**Figure 4:**
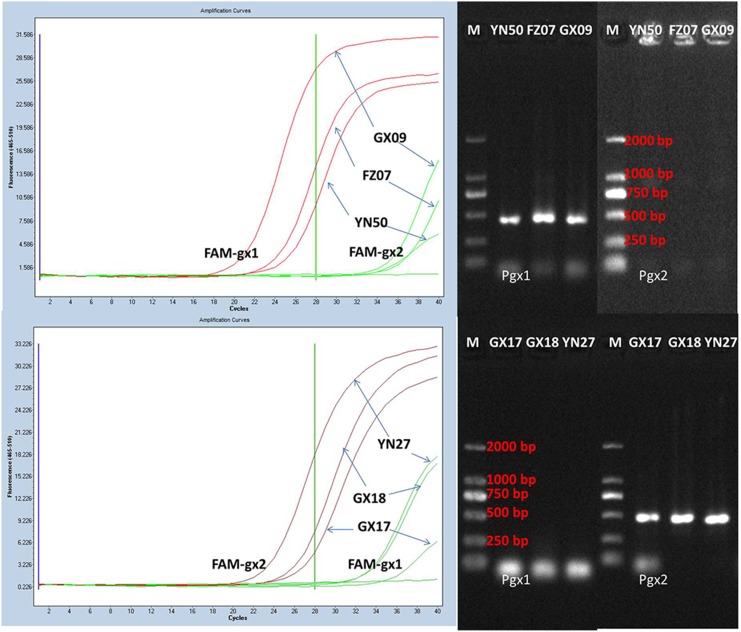
Specificity of primers and probes for detection of *F. verticillioides* (A: FAM-gx1 and Pgx1), and for detection of *F. proliferatum* (B: FAM-gx2 and Pgx2) using multiple primer sets. (A) Templates were fungal DNA from *F. verticillioides* isolates (YN50, FZ07 and GX09). (B) Templates were fungal DNA from *F. proliferatum* isolates (GX17, GX18 and YN27). M: DL2000 (Takara, Dalian).

TaqMan PCR assays using both FAM-gx1 and FAM-gx2 primer-probe combinations simultaneously detected the pokkah boeng pathogen in the isolates of *F. verticillioides* (GX09, FZ07 and YN50) using FAM-gx1 or in the isolates of *F. proliferatum* (GX17, GX18 and YN27) using FAM-gx2. Conventional PCR assays also yielded similar positive results (Fig. 4).

**Figure 5:**
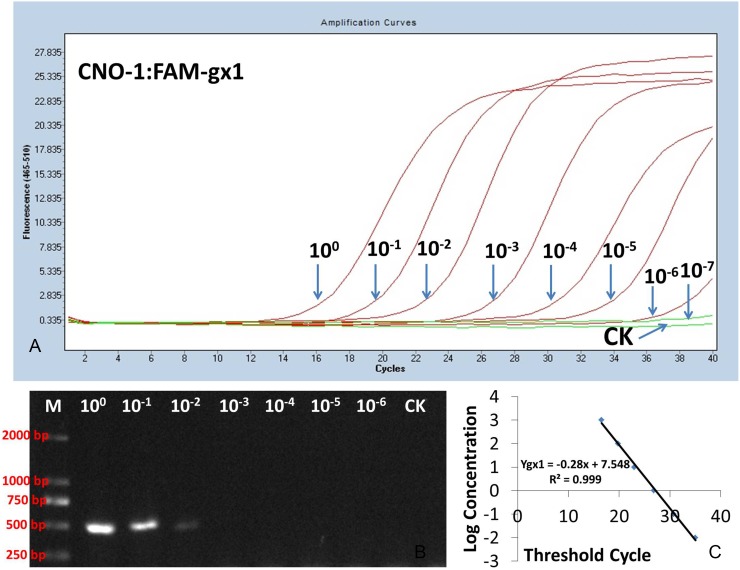
Sensitivity of the species-specific PCR for detection of *F. verticillioides* using serial dilutions of fungal DNA extracted from CNO-1. TaqMan PCR amplification of the FAM-gx1 primer–probe combination; (B) conventional PCR; (C) A standard curve obtained in TaqMan PCR with the FAM-gx1 primer–probe combination using serial dilutions of fungal DNA extracted from CNO-1 (isolate of *F. verticillioides*). Templates were serial dilutions (10^0^∼10^−7^) of fungal DNA (1,000 ng/µl) extracted from isolate CNO-1.

**Figure 6:**
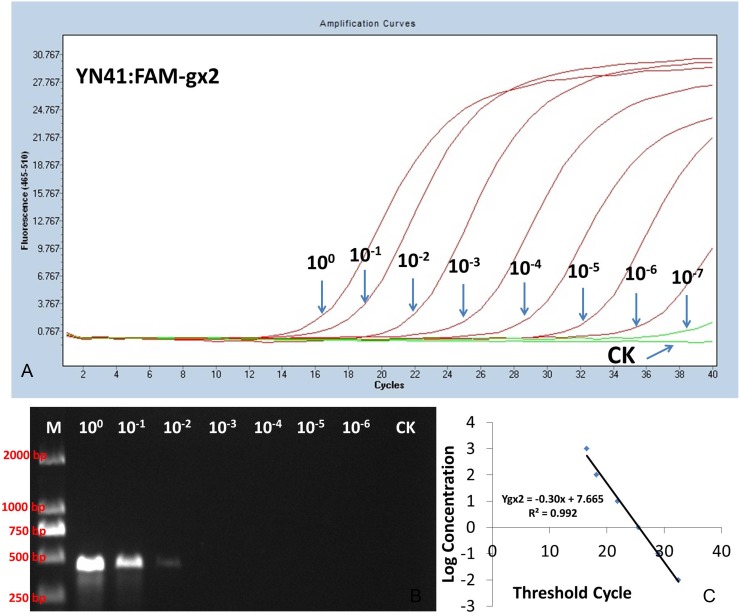
Sensitivity of the species-specific PCR for detection of *F. proliferatum* using serial dilutions of fungal DNA extracted from YN-41. TaqMan PCR amplification of the FAM-gx2 primer–probe combination; (B) conventional PCR of Pgx2; (C) A standard curve obtained in TaqMan PCR with the FAM-gx2 primer–probe combination using serial dilutions of fungal DNA extracted from YN-41 (isolate of *F. proliferatum*). Templates were serial dilutions (10^0^∼10^−7^) of fungal DNA (1,000 ng/µl) extracted from isolate YN-41.

Serial dilutions of DNA extracted from the isolates of *F. verticillioides* (CNO-1) and *F. proliferatum* (YN-41) were used to evaluate the assay sensitivity of TaqMan PCR and conventional PCR. TaqMan PCR detected *F. verticillioides* (CNO-1) and *F. proliferatum* (YN-41) in 1 µl of the 10^−5^ dilution of DNA (10 pg: 1,000 ng/µl×10^−5^×1 µl) (Fig. 4, Fig. 5). However, for the conventional PCR assay, the primer sets (Pgx1 or Pgx2) only detected *F. verticillioides* (CNO-1) and *F. proliferatum* (YN-41) in 1 µl of the 10^−2^ dilution (10 ng: 1000 ng/µl×10^−2^×1 µl) (Fig. 5, Fig. 6). These results showed that the detection limit of TaqMan PCR was 1,000-fold over that of the conventional PCR.

### 3.3 Pathogen detection for sugarcane pokahh boeng in field-grown leaves

DNA samples from 28 pokkah boeng symptomatic and asymptomatic leaves were detected by multiplex TaqMan PCR (Table 1). All five asymptomatic sugarcane leaves tested negative for *F. verticillioides* or *F. proliferatum* by both conventional PCR and TaqMan PCR. Twenty-two out of 23 symptomatic samples (95.7%) tested positive using the species-specific FAM-gx1 for *F. verticillioides*, of which the Ct values ranged from 19.9 to 30.8. However, only seven (30.4%) tested positive using the species-specific FAM-gx2 for *F. proliferatum* (Ct values ranged from 25.9 to 29.9), whereas they were also positive for *F. verticillioides* (Ct values ranged from 21.6 to 30.8), implying that all 7 samples were infected by both *F. proliferatum* and *F. verticillioides*, while the other 15 gx1-positive samples were only infected by *F. verticillioides.* For conventional PCR, only one sample (S4) tested positive using the Pgx1 for *F. verticillioides*. All of the other samples tested negative by both primers (Pgx1 and Pgx2). These results also indicated that TaqMan PCR assay was more sensitive in detecting sugarcane pokkah boeng compared to the conventional PCR, particularly for the leaf samples.

## Discussion

Pokkah boeng, one of the most serious fungal diseases in most, if not all, sugarcane-producing areas of the world [22], causes yield losses in sugarcane, ranging from 40% to 60% in the susceptible cultivars [23]. It is caused by several species of *Fusarium*, including *F. verticillioides* (*F. moniliforme*) [24], [25], *F. sacchari* [26], *F. proliferatum,* and *F. subglutinans* [27], but no causal agent was established in China. Early and efficient identification of this pathogen is therefore important to establish the pokkah boeng management strategy in the sugarcane industry.

In the present study, sugarcane plants affected by pokkah boeng were collected from the major sugarcane producing areas (Guangxi, Yunnan, Guangdong, Fujian, Hainan) in China throughout 2012 and 2013. A total of 101 isolates were recovered from these diseased samples. The results from the morphological observation and the phylogenetic tree of rDNA-ITS region sequence amplified using fungus-conserved ITS1 and ITS4 primers indicated that more than 90% of the isolates (94 isolates) belonged to *F. verticillioides,* which was closely related to *F. sacchari*, while only 7% of the isolates (7 isolates) belonged to *F. proliferatum,* which was closely related to *F. fujikuroi.* Abundant microconidia of *F. verticillioides* with sizes ranging from 7.1–13.4×0.5–2.1 µm were formed in false heads and absent in the chain, which differentiated from those of *F. fujikuroi* and *F. proliferatum* [13]. *F. proliferatum* and *F. fujikuroi* were closely related species to one another [12] and were difficult to identify based solely on morphology [13]. *F. fujikuroi* was found primarily on rice in Asia where it caused the bakanae disease through the overproduction of gibberellic acid [28]. *F. proliferatum* was widespread and occurred on hosts ranging from rice to corn and sorghum, but it was usually not the dominant pathogen [29], [30].

PCR-based methods offer rapid and cost-effective tools of molecular diagnosis. Real-time PCR coupled with conventional PCR techniques can reliably detect and quantify pathogens in the fungal isolates or from infected leaf samples [9]. Several PCR assays have been developed to identify species of *Fusarium* based on the translation elongation factor gene (*tef1*) [31]–[33], polyketide synthase gene (*pks*) [34], and trichodiene synthase gene (*tri5*) [35]. The sequence of the ITS-rDNA is highly conserved but sufficiently variable among species of *Fusarium* species complex. The ability of the ITS region to differentiate and provide accurate and rapid detection of fungus has been reported at the species-level [36]. Specific PCR primers based on ITS sequences for *Fusarium* species complex have been designed based on two single nucleotide polymorphisms (SNPs) present in the ITS region [37]. In this study, we presented a rapid, efficient, and specific PCR assay to detect pathogens of sugarcane pokkah boeng based on the differences in the ITS-rDNA sequences between two species of *F. verticillioides* and *F. proliferatum*. Species-specific primers and probes were designed and their specificity and sensitivity were evaluated to detect *F. verticillioides* and *F. proliferatum* using purified DNA from 84 isolates or 28 infected sugarcane leaves. TaqMan PCR using FAM-gx1 and FAM-gx2 primer–probe combinations was very sensitive, with a detection limit of 10 pg/µl fungal DNA. Conventional PCR using Pgx1 and Pgx2 primers, which also targeted the ITS-rDNA region, resulted in fragments at the size of 439 bp with Pgx1 and 400 bp with Pgx2. However, the detection limit for the conventional PCR was approximately 10 ng of fungal DNA input. These results indicated that the TaqMan PCR was approximately 1,000-fold more sensitive than the conventional PCR for detection of *F. verticillioides* and *F. proliferatum*. Furthermore, the conventional PCR failed to detect *F. verticillioides* and *F. proliferatum* in the field-grown, infected cane plants that could be detected using the TaqMan PCR. Use of the TaqMan PCR with total plant genomic DNA extracted from symptomatic samples of pokkah boeng demonstrated that inhibitors or other substances from sugarcane did not affect the results of this assay. The sensitivity of our multiplex TaqMan PCR in the detection and identification of *F. verticillioides* and *F. proliferatum* was not affected by a positive internal control reaction or by the presence of both targets of *F. verticillioides* and *F. proliferatum*. Therefore, in comparison to the TaqMan PCR, the amount of fungal DNA extracted from leaf samples was a limiting factor to directly detect sugarcane pokkah boeng using conventional PCR.

TaqMan PCR amplifications from the field-grown samples showed that 22 out of 23 (95.6%) symptomatic sugarcane leaf samples tested positive for *F. verticillioides* by FAM-gx1. Only seven samples (30.4%) tested positive for *F. proliferatum* by FAM-gx2, which were also positive for *F. verticillioides* by FAM-gx1. Two isolates of *F. verticillioides* (HC24-1 and HC24-2) and one isolate of *F. proliferatum* (HC24-3) were recovered from the same sample collected in Hechi, Guangxi, which indicated the mixed infection of *F. verticillioides* and *F. proliferatum.* Only one symptomatic leaf sample (S21) tested negative in all PCR assays. The sample S21 had symptoms similar to sugarcane pokkah boeng, such as the twisted and curling symptoms of crown leaves (Fig. S2), and was found to be infected by *Phoma* sp. based on the morphological characteristics and the ITS sequence of rDNA [38]. This further confirmed that pathogen detection based solely on symptoms was not sufficient for accurate diagnosis of plant disease. Sample S4, with the lowest Ct value of 18, indicated that some cane tissues could support a high pathogen load. However, the pokkah boeng pathogen could not be detected in most of leaf tissues by conventional PCR and required the more sensitive TaqMan PCR.

In conclusion, two pathogens (*F. verticillioides* and *F. proliferatum*) to cause sugarcane pokahh boeng in China were identified by morphological observation, pathogenicity test, and phylogenetic analysis. Both conventional PCR and TaqMan PCR were developed to reliably detect the species of *Fusarium* using purified fungal DNA as input based on their ITS sequence. The TaqMan PCR was a more efficient tool for the early diagnosis of disease, and it was capable of detecting pokkah boeng in symptomatic young tissues of infected plants. This technique promises to be a valuable component in large-scale diagnosis and management as shown by current use in the ongoing detection of pokkah boeng in field surveys. To the best of our knowledge, this is the first report to detect and identify *F. verticillioides* and *F. proliferatum*, which cause pokkah boeng disease in China.

## Supporting Information

Figure S1 **Geographical locations where Pokkah boeng surveys were conducted in the major sugarcane growing areas of People’s Republic of China (PRC).** The map was drawn using DIVA-GIS software based on coordinates recorded for each locality with a GPS device. ▴: isolates of *Fusarium* species complex; ▪: isolates of *F. fujikuroi;* *: isolates of *Sporisorium scitamineum; †:* isolates of *Phoma* sp.; •: isolates of *Acremonium* sp.
(TIF)

Figure S2 **Asymptomatic (As) and symptomatic (S) field-grown sugarcane plants.** Asymptomatic (As1) and symptomatic (S2, S3, S4 and S7) samples were collected from a field plot at Guangxi University; Photographs of pokahh boeng-like symptoms (S14 and S21) were taken in a field at Chongzuo Agriculture Experimental Station.
(TIF)

Table S1 Isolates used for phylogenetic analyses and their corresponding GenBank accession numbers.
(DOCX)
