# Supplementary figures and images for: Species-Specific Detection and Identification of Fusarium Species Complex, the Causal Agent of Sugarcane Pokkah Boeng in China
Source: PLoS One. 2014 Aug 20;9(8):e104195. doi: 10.1371/journal.pone.0104195 (PMC4139266; doi:10.1371/journal.pone.0104195)

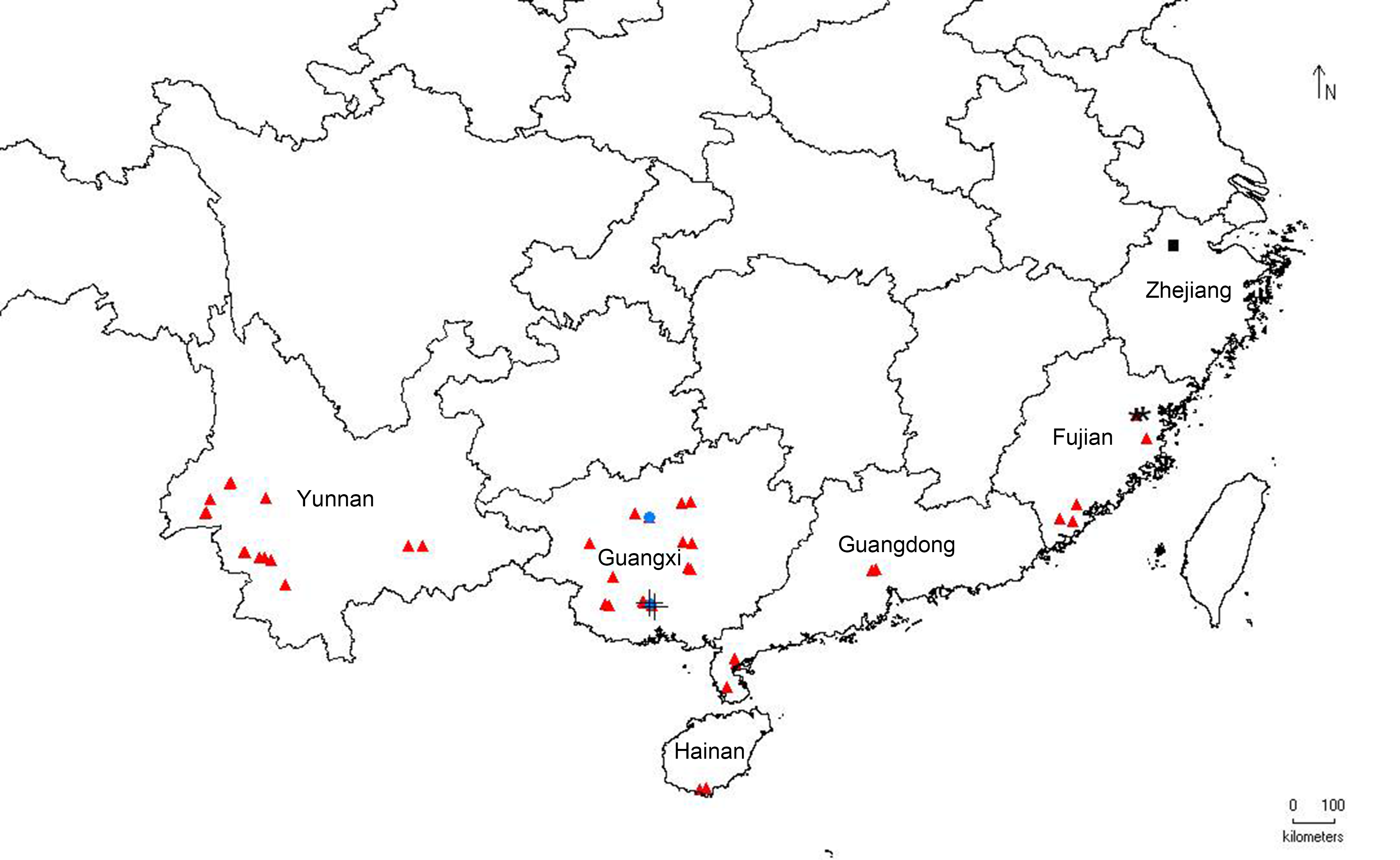

Supplement: Figure S1 — Geographical locations where Pokkah boeng surveys were conducted in the major sugarcane growing areas of People’s Republic of China (PRC). The map was drawn using DIVA-GIS software based on coordinates recorded for each locality with a GPS device. ▴: isolates of Fusarium species complex; ▪: isolates of F. fujikuroi; *: isolates of Sporisorium scitamineum; †: isolates of Phoma sp.; •: isolates of Acremonium sp. (TIF) [file pone.0104195.s001.tif]

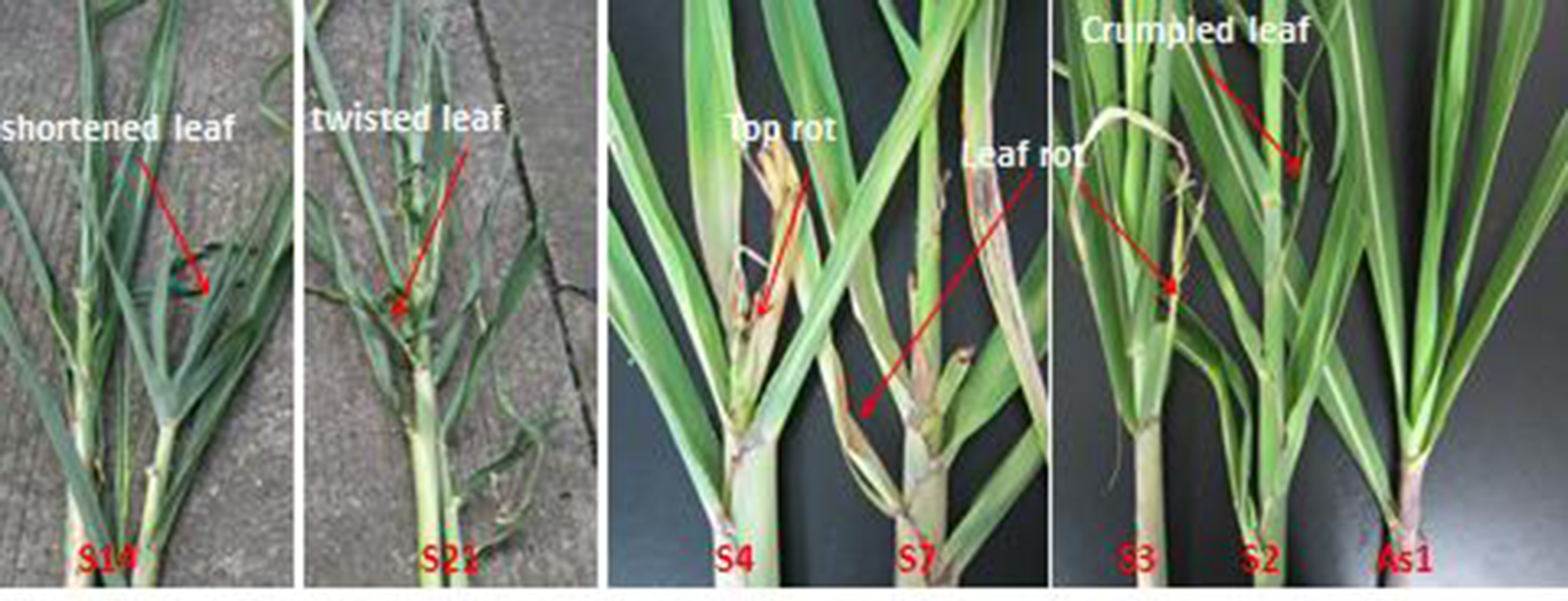

Supplement: Figure S2 — Asymptomatic (As) and symptomatic (S) field-grown sugarcane plants. Asymptomatic (As1) and symptomatic (S2, S3, S4 and S7) samples were collected from a field plot at Guangxi University; Photographs of pokahh boeng-like symptoms (S14 and S21) were taken in a field at Chongzuo Agriculture Experimental Station. (TIF) [file pone.0104195.s002.tif]
