# Supplementary material for: Species-Specific Detection and Identification of Fusarium Species Complex, the Causal Agent of Sugarcane Pokkah Boeng in China
Source: PLoS One. 2014 Aug 20;9(8):e104195. doi: 10.1371/journal.pone.0104195 (PMC4139266; doi:10.1371/journal.pone.0104195)
Supplement: Table S1 — Isolates used for phylogenetic analyses and their corresponding GenBank accession numbers. (DOCX) [file pone.0104195.s003.docx]

**Table S1 Isolates used for phylogenetic analyses and their corresponding GenBank accession numbers.**

| **Isolates** | ***Species*** | **Phylogenetic groups** | **Hosts** | **Geographic origin** | **Genbank no.** |
| --- | --- | --- | --- | --- | --- |
| DH02 | *F. verticillioides* | gx1 | Sugarcane | Dehong,Yunnan | KJ629515 |
| DH03 | *F. verticillioides* | gx1 | Sugarcane | Dehong,Yunnan | KJ629468 |
| DH04 | *F. verticillioides* | gx1 | Sugarcane | Dehong,Yunnan | KJ629469 |
| DH05 | *F. verticillioides* | gx1 | Sugarcane | Dehong,Yunnan | KJ629470 |
| DH06 | *F. verticillioides* | gx1 | Sugarcane | Dehong,Yunnan | KJ629471 |
| DH08 | *F. verticillioides* | gx1 | Sugarcane | Dehong,Yunnan | KJ629472 |
| DH09 | *F. verticillioides* | gx1 | Sugarcane | Dehong,Yunnan | KJ629473 |
| DH11 | *F. verticillioides* | gx1 | Sugarcane | Dehong,Yunnan | KJ629474 |
| DH12 | *F. verticillioides* | gx1 | Sugarcane | Dehong,Yunnan | KJ629475 |
| DH13 | *F. verticillioides* | gx1 | Sugarcane | Dehong,Yunnan | KJ629476 |
| DH16 | *F. verticillioides* | gx1 | Sugarcane | Dehong,Yunnan | KJ629477 |
| DH17 | *F. verticillioides* | gx1 | Sugarcane | Dehong,Yunnan | KJ629478 |
| DH19 | *F. verticillioides* | gx1 | Sugarcane | Dehong,Yunnan | KJ629479 |
| DH24 | *F. verticillioides* | gx1 | Sugarcane | Dehong,Yunnan | KJ629480 |
| YN25 | *F. verticillioides* | gx1 | Sugarcane | Dehong,Yunnan | KJ629482 |
| DH28 | *F. verticillioides* | gx1 | Sugarcane | Dehong,Yunnan | KJ629483 |
| DH29 | *F. verticillioides* | gx1 | Sugarcane | Dehong,Yunnan | KJ629484 |
| DH30 | *F. verticillioides* | gx1 | Sugarcane | Baoshan,Yunnan | KJ629485 |
| BS32 | *F. verticillioides* | gx1 | Sugarcane | Baoshan,Yunnan | KJ629505 |
| BS33 | *F. verticillioides* | gx1 | Sugarcane | Baoshan,Yunnan | KJ629506 |
| BS34 | *F. verticillioides* | gx1 | Sugarcane | Baoshan,Yunnan | KJ629507 |
| BS35 | *F. verticillioides* | gx1 | Sugarcane | Baoshan,Yunnan | KJ629508 |
| BS37 | *F. verticillioides* | gx1 | Sugarcane | Baoshan,Yunnan | KJ629509 |
| BS38 | *F. verticillioides* | gx1 | Sugarcane | Baoshan,Yunnan | KJ629510 |
| BS39 | *F. verticillioides* | gx1 | Sugarcane | Baoshan,Yunnan | KJ629511 |
| BS40 | *F. verticillioides* | gx1 | Sugarcane | Baoshan,Yunnan | KJ629512 |
| BS42 | *F. verticillioides* | gx1 | Sugarcane | Baoshan,Yunnan | KJ629514 |
| SJ44 | *F. verticillioides* | gx1 | Sugarcane | Shuangjiang,Yunnan | KJ629493 |
| SJ45 | *F. verticillioides* | gx1 | Sugarcane | Shuangjiang,Yunnan | KJ629494 |
| SJ46 | *F. verticillioides* | gx1 | Sugarcane | Shuangjiang,Yunnan | KJ629495 |
| SJ47 | *F. verticillioides* | gx1 | Sugarcane | Shuangjiang,Yunnan | KJ629496 |
| SJ48 | *F. verticillioides* | gx1 | Sugarcane | Shuangjiang,Yunnan | KJ629497 |
| SJ49 | *F. verticillioides* | gx1 | Sugarcane | Shuangjiang,Yunnan | KJ629498 |
| YN50 | *F. verticillioides* | gx1 | Sugarcane | Shuangjiang,Yunnan | KJ629499 |
| SJ51 | *F. verticillioides* | gx1 | Sugarcane | Shuangjiang,Yunnan | KJ629500 |
| GM53 | *F. verticillioides* | gx1 | Sugarcane | Gengma,Yunnan | KJ629486 |
| YN54 | *F. verticillioides* | gx1 | Sugarcane | Gengma,Yunnan | KJ629487 |
| GM55 | *F. verticillioides* | gx1 | Sugarcane | Gengma,Yunnan | KJ629488 |
| GM56 | *F. verticillioides* | gx1 | Sugarcane | Gengma,Yunnan | KJ629489 |
| GM57 | *F. verticillioides* | gx1 | Sugarcane | Gengma,Yunnan | KJ629490 |
| GM58 | *F. verticillioides* | gx1 | Sugarcane | Gengma,Yunnan | KJ629491 |
| SJ62 | *F. verticillioides* | gx1 | Sugarcane | Shuangjiang,Yunnan | KJ629501 |
| GM64 | *F. verticillioides* | gx1 | Sugarcane | Gengma,Yunnan | KJ629492 |
| SJ65-2 | *F. verticillioides* | gx1 | Sugarcane | Shuangjiang,Yunnan | KJ629502 |
| SJ65 | *F. verticillioides* | gx1 | Sugarcane | Shuangjiang,Yunnan | KJ629503 |
| SJ67 | *F. verticillioides* | gx1 | Sugarcane | Shuangjiang,Yunnan | KJ629504 |
| CT46 | *F. verticillioides* | gx1 | Sugarcane | Changtai,Fujian | KJ629516 |
| HC01 | *F. verticillioides* | gx1 | Sugarcane | Hechi,Guangxi | KJ629517 |
| GX08 | *F. verticillioides* | gx1 | Sugarcane | Hechi,Guangxi | KJ629518 |
| HC11 | *F. verticillioides* | gx1 | Sugarcane | Hechi,Guangxi | KJ629519 |
| GX12 | *F. verticillioides* | gx1 | Sugarcane | Hechi,Guangxi | KJ629520 |
| HC13 | *F. verticillioides* | gx1 | Sugarcane | Hechi,Guangxi | KJ629521 |
| HC20 | *F. verticillioides* | gx1 | Sugarcane | Hechi,Guangxi | KJ629523 |
| HC24-1 | *F. verticillioides* | gx1 | Sugarcane | Hechi,Guangxi | KJ629524 |
| HC24-2 | *F. verticillioides* | gx1 | Sugarcane | Hechi,Guangxi | KJ629525 |
| HC30 | *F. verticillioides* | gx1 | Sugarcane | Hechi,Guangxi | KJ629527 |
| HC34 | *F. verticillioides* | gx1 | Sugarcane | Hechi,Guangxi | KJ629529 |
| HC35 | *F. verticillioides* | gx1 | Sugarcane | Hechi,Guangxi | KJ629530 |
| GX28 | *F. verticillioides* | gx1 | Sugarcane | Laibing,Guangxi | KJ629531 |
| LC04 | *F. verticillioides* | gx1 | Sugarcane | Liucheng,Guangxi | KJ629532 |
| LC05 | *F. verticillioides* | gx1 | Sugarcane | Liucheng,Guangxi | KJ629533 |
| LC07 | *F. verticillioides* | gx1 | Sugarcane | Liucheng,Guangxi | KJ629534 |
| GX09 | *F. verticillioides* | gx1 | Sugarcane | Liucheng,Guangxi | KJ629535 |
| LC10 | *F. verticillioides* | gx1 | Sugarcane | Liucheng,Guangxi | KJ629536 |
| LC15 | *F. verticillioides* | gx1 | Sugarcane | Liucheng,Guangxi | KJ629537 |
| LCA9 | *F. verticillioides* | gx1 | Sugarcane | Liucheng,Guangxi | KJ629539 |
| LW50 | *F. verticillioides* | gx1 | Sugarcane | Zhangzhou,Fujian | KJ629540 |
| LW51 | *F. verticillioides* | gx1 | Sugarcane | Zhangzhou,Fujian | KJ629541 |
| LW54 | *F. verticillioides* | gx1 | Sugarcane | Zhangzhou,Fujian | KJ629542 |
| LW60 | *F. verticillioides* | gx1 | Sugarcane | Zhangzhou,Fujian | KJ629543 |
| LW64 | *F. verticillioides* | gx1 | Sugarcane | Zhangzhou,Fujian | KJ629544 |
| LW65 | *F. verticillioides* | gx1 | Sugarcane | Zhangzhou,Fujian | KJ629545 |
| LW67 | *F. verticillioides* | gx1 | Sugarcane | Zhangzhou,Fujian | KJ629546 |
| GX41 | *F. verticillioides* | gx1 | Sugarcane | Nanning,Guangxi | KJ629547 |
| FZ03 | *F. verticillioides* | gx1 | Sugarcane | Fuzhou,Fujian | KJ629550 |
| FZ04 | *F. verticillioides* | gx1 | Sugarcane | Fuzhou,Fujian | KJ629551 |
| FZ06 | *F. verticillioides* | gx1 | Sugarcane | Fuzhou,Fujian | KJ629552 |
| FZ07 | *F. verticillioides* | gx1 | Sugarcane | Fuzhou,Fujian | KJ629553 |
| FZ08 | *F. verticillioides* | gx1 | Sugarcane | Fuzhou,Fujian | KJ629554 |
| FZ09 | *F. verticillioides* | gx1 | Sugarcane | Fuzhou,Fujian | KJ629555 |
| FZ10 | *F. verticillioides* | gx1 | Sugarcane | Fuzhou,Fujian | KJ629556 |
| FZ11 | *F. verticillioides* | gx1 | Sugarcane | Fuzhou,Fujian | KJ629557 |
| FZ12 | *F. verticillioides* | gx1 | Sugarcane | Fuzhou,Fujian | KJ629558 |
| FZ13 | *F. verticillioides* | gx1 | Sugarcane | Fuzhou,Fujian | KJ629559 |
| FZ14 | *F. verticillioides* | gx1 | Sugarcane | Fuzhou,Fujian | KJ629560 |
| FZ15 | *F. verticillioides* | gx1 | Sugarcane | Fuzhou,Fujian | KJ629561 |
| FZ42 | *F. verticillioides* | gx1 | Sugarcane | Fuzhou,Fujian | KJ629562 |
| FZ46 | *F. verticillioides* | gx1 | Sugarcane | Fuzhou,Fujian | KJ629563 |
| CNO-1 | *F. verticillioides* | gx1 | Sugarcane | Chongzuo,Guangxi | KJ629564 |
| 00-122 | *F. verticillioides* | gx1 | Sugarcane | Chongzuo,Guangxi | KJ629565 |
| 02-901 | *F. verticillioides* | gx1 | Sugarcane | Zhanjiang,Guangdong | KJ629566 |
| FN22 | *F. verticillioides* | gx1 | Sugarcane | Zhanjiang,Guangdong | KJ629567 |
| FN28-2 | *F. verticillioides* | gx1 | Sugarcane | Zhanjiang,Guangdong | KJ629569 |
| FN29 | *F. verticillioides* | gx1 | Sugarcane | Zhanjiang,Guangdong | KJ629570 |
| YN27 | *F. proliferatum* | gx2 | Sugarcane | Dehong,Yunnan | KJ629482 |
| YN41 | *F. proliferatum* | gx2 | Sugarcane | Baoshan,Yunnan | KJ629513 |
| GX17 | *F. proliferatum* | gx2 | Sugarcane | Hechi,Guangxi | KJ629522 |
| HC24-3 | *F. proliferatum* | gx2 | Sugarcane | Hechi,Guangxi | KJ629526 |
| HC31 | *F. proliferatum* | gx2 | Sugarcane | Hechi,Guangxi | KJ629528 |
| GX18 | *F. proliferatum* | gx2 | Sugarcane | Liucheng,Guangxi | KJ629538 |
| EM-1-10 | *F. proliferatum* | gx2 | Rice | Hangzhou,Zhejiang | KJ629548 |
| EM-1-17 | *F. proliferatum* | gx2 | Rice | Hangzhou,Zhejiang | KJ629549 |
| FN28 | *F. proliferatum* | gx2 | Sugarcane | Fuzhou,Fujian | KJ629568 |
| CBS 221.76 | *F. fujikuroi* | gx2 | Rice | [Netherlands](http://www.cbs.knaw.nl/Collections/BioloMICS.aspx?Link=T&TableKey=14682616000000019&Rec=45&Fields=All) | X94176 |
| 798A | *F. sacchari* | gx1 | Sugarcane | Iran | JQ363733 |
| 8044 | *F. sacchari* | gx1 | Sugarcane | Iran | JQ363734 |
| 77R | *F. proliferatum* | gx2 | Sugarcane | Iran | JQ363736 |
| Fs-14 | *F. sacchari* | gx1 | Sugarcane | India | AB374091 |
| Fs-13 | *F. sacchari* | gx1 | Sugarcane | India | AB374090 |
| Fs-12 | *F. sacchari* | gx1 | Sugarcane | India | AB374089 |
| Fs-11 | *F. sacchari* | gx1 | Sugarcane | India | AB374088 |
| FproStRIN1 | *F. proliferatum* | gx2 | Rice | India | KC577192 |
| 297a-09 | *F. proliferatum* | gx2 | Sorghum | Serbia | JQ412110 |
| M86032 | *Fusarium sp.* | gx1 | Sugarcane | India | EF680760 |
| UP0121 | *Fusarium sp.* | gx1 | Sugarcane | India | EF680759 |
| B03176 | *Fusarium sp.* | gx1 | Sugarcane | India | EF680758 |
| TN2-86032 | *Fusarium sp.* | gx1 | Sugarcane | India | EF680757 |
| P89003 | *Fusarium sp.* | gx2 | Sugarcane | India | EF680754 |
| TN186032 | *Fusarium sp.* | gx1 | Sugarcane | India | EF680755 |
| H89003 | *Fusarium sp.* | gx1 | Sugarcane | India | EF680756 |
| NRRL 13383 | *F. graminearum* | gx1 | Corn | [Iran](http://www.cbs.knaw.nl/Collections/BioloMICS.aspx?Link=T&TableKey=14682616000000019&Rec=141&Fields=All) | [U85536](http://www.cbs.knaw.nl/Collections/BioloMICS.aspx?Link=T&TargetKey=14682616000000137&Rec=12796102&Revert=F) |
| NRRL 13999 | *F. sacchari* | gx1 | Sugarcane | India | [U34556](http://www.cbs.knaw.nl/Fusarium/BioloMICS.aspx?Link=T&TargetKey=1037978000000131&Rec=1793&Revert=F) |
| NRRL 22172 | *F. verticillioides* | gx1 | Corn | Germany | [U34555](http://www.cbs.knaw.nl/Fusarium/BioloMICS.aspx?Link=T&TargetKey=1037978000000131&Rec=1876&Revert=F) |
